# Supplementary material for: Undernutrition, Sarcopenia, and Frailty in Fragility Hip Fracture: Advanced Strategies for Improving Clinical Outcomes
Source: Nutrients. 2020 Dec 4;12(12):3743. doi: 10.3390/nu12123743 (PMC7762043; doi:10.3390/nu12123743)
Supplement: Supplementary file 1 [file nutrients-12-03743-s001.pdf]

Supplementary Materials

Table S1. Results of the quality assessment of the study on undernutrition and clinical outcomes.

Author	Criteria 1	Criteria 2	Criteria 3	Criteria 4	Criteria 5	Criteria 6	Criteria 7	Criteria 8	Criteria 9	Criteria 10	Criteria 11	Criteria 12	Criteria 13	Criteria 14	Quality Rating
Miyanishi K et al. (2010)	Y	Y	NR	Y	N	Y	Y	NA	Y	NA	Y	NR	NR	N	Good
Koren-Hakim T et al. (2012)	Y	Y	Y	Y	N	Y	Y	NA	Y	NA	Y	NR	NR	N	Good
Gumieiro DN et al. (2012)	Y	Y	NR	Y	Y	Y	Y	NA	Y	NA	Y	NR	Y	N	Good
Drevet S et al. (2014)	Y	N	NR	Y	N	Y	Y	NA	Y	NA	N	NR	NA	N	Poor
Goisser S et al. (2015)	Y	Y	Y	Y	N	Y	Y	NA	Y	NA	Y	NR	Y	N	Good
Bajada S et al. (2015)	Y	Y	Y	Y	N	Y	Y	NA	Y	NA	Y	NR	N	N	Good
van Wissen J et al. (2016)	Y	Y	Y	Y	N	Y	Y	NA	Y	NA	Y	NR	Y	N	Fair
Miu KYD et al. (2017)	Y	Y	NR	Y	N	Y	Y	NA	Y	NA	Y	NR	NR	N	Poor
Helminen H et al. (2017)	Y	Y	Y	Y	N	Y	Y	NA	Y	NA	Y	NR	Y	N	Good
Vosoughi AR et al. (2017)	Y	Y	NR	Y	N	Y	Y	NA	Y	NA	Y	NR	N	N	Fair
Mazzola P et al. (2017)	Y	Y	Y	Y	N	Y	Y	NA	Y	NA	Y	NR	Y	N	Good
Inoue T et al. (2017)	Y	Y	Y	Y	N	Y	Y	NA	Y	NA	Y	NR	Y	Y	Good
Nishioka S et al. (2018)	Y	Y	NR	Y	Y	NA	NA	NA	Y	Y	Y	NR	Y	Y	Good
Stone AV et al. (2018)	Y	Y	NR	Y	N	Y	Y	NA	Y	NA	Y	NR	NR	N	Fair
Zanetti M et al. (2018)	Y	Y	Y	Y	N	Y	Y	NA	Y	NA	Y	NR	Y	N	Good
Kotera A et al. (2019)	Y	Y	Y	Y	N	Y	Y	NA	Y	NA	Y	NR	Y	N	Good
Yagi T et al. (2020)	Y	Y	NR	Y	N	Y	Y	NA	Y	NA	Y	NR	NR	N	Fair
Hao L et al. (2020)	Y	Y	Y	Y	N	Y	Y	NA	Y	NA	Y	NR	Y	N	Good
Han TS et al. (2020)	Y	Y	NR	Y	Y	Y	Y	NA	Y	NA	Y	NR	Y	N	Good

Quality of the selected observational study was assessed using the National Institutes of Health (NIH) Quality Assessment tool for Observational Cohort and Cross-Sectional Studies. Criteria1. Was the research question or objective in this paper clearly stated? Criteria 2. Was the study population clearly specified and defined? Criteria 3. Was the participation rate of eligible persons at least 50%? Criteria 4. Were all the subjects selected or recruited from the same or similar populations (including the same time period)? Were inclusion and exclusion

criteria for being in the study prespecified and applied uniformly to all participants? Criteria 5. Was a sample size justification, power description, or variance and effect estimates provided? Criteria 6. For the analyses in this paper, were the exposure(s) of interest measured prior to the outcome(s) being measured? Criteria 7. Was the timeframe sufficient so that one could reasonably expect to see an association between exposure and outcome if it existed? Criteria 8. For exposures that can vary in amount or level, did the study examine different levels of the exposure as related to the outcome (e.g., categories of exposure, or exposure measured as continuous variable)? Criteria 9. Were the exposure measures (independent variables) clearly defined, valid, reliable, and implemented consistently across all study participants? Criteria 10. Was the exposure(s) assessed more than once over time? Criteria 11. Were the outcome measures (dependent variables) clearly defined, valid, reliable, and implemented consistently across all study participants? Criteria 12. Were the outcome assessors blinded to the exposure status of participants? Criteria 13. Was loss to follow-up after baseline 20% or less? Criteria 14. Were key potential confounding variables measured and adjusted statistically for their impact on the relationship between exposure(s) and outcome(s)? The overall judgment is determined by Good, Fair, Poor. CD, cannot determine; NA, not applicable; NR, not reported.

Table S2. Results of the quality assessment of the study on sarcopenia and clinical outcomes.

Author	Criteria 1	Criteria 2	Criteria 3	Criteria 4	Criteria 5	Criteria 6	Criteria 7	Criteria 8	Criteria 9	Criteria 10	Criteria 11	Criteria 12	Criteria 13	Criteria 14	Quality Rating
González-Montalvo JI et al. (2015)	Y	Y	Y	Y	N	Y	Y	NA	Y	NA	Y	NR	Y	N	Good
Di Monaco M et al. (2015)	Y	Y	NR	Y	N	Y	Y	NA	Y	NA	Y	NR	Y	N	Good
Landi F et al. (2017)	Y	Y	Y	Y	N	Y	Y	NA	Y	NA	Y	NR	Y	N	Good
Chang C Di et al. (2018)	Y	Y	NR	Y	N	Y	Y	NA	Y	NA	Y	NR	Y	N	Good
Kim YK et al. (2018)	Y	Y	N	Y	N	Y	Y	NA	Y	NA	Y	NR	Y	N	Poor
Yoo J Il et al. (2018)	Y	Y	Y	Y	N	Y	Y	NA	Y	NA	N	NR	Y	N	Poor
Steihaug OM et al. (2018)	Y	Y	N	Y	N	Y	Y	NA	Y	NA	Y	NR	Y	N	Fair
Malafarina V et al. (2019)	Y	Y	Y	Y	N	Y	Y	NA	Y	Y	Y	NR	Y	N	Good
Byun SE et al. (2019)	Y	Y	Y	Y	N	Y	Y	NA	Y	NA	Y	NR	Y	N	Good
Chen YP et al. (2020)	Y	Y	Y	Y	N	Y	Y	NA	Y	NA	Y	NR	N	N	Fair
Chiles Shaffer N et al. (2020)	Y	Y	Y	Y	N	Y	Y	NA	Y	NA	Y	NR	N	N	Good
Shin WC et al. (2020)	Y	Y	Y	Y	N	Y	Y	NA	Y	NA	Y	NR	Y	N	Good
Nagano A et al. (2020)	Y	Y	Y	Y	N	Y	Y	NA	Y	NA	Y	NR	Y	Y	Good
Ha YC et al. (2020)	Y	Y	Y	Y	N	N	NA	NA	Y	NA	NA	NR	NA	N	Fair

Quality of the selected observational study was assessed using the National Institutes of Health (NIH) Quality Assessment tool for Observational Cohort and Cross-Sectional Studies. Criteria1. Was the research question or objective in this paper clearly stated? Criteria 2. Was the study population clearly specified and defined? Criteria 3. Was the participation rate of eligible persons at least 50%? Criteria 4. Were all the subjects selected or recruited from the same or similar populations (including the same time period)? Were inclusion and exclusion criteria for being in the study prespecified and applied uniformly to all participants? Criteria 5. Was a sample size justification, power description, or variance and effect estimates provided? Criteria 6. For the analyses in this paper, were the exposure(s) of interest measured prior to the outcome(s) being measured? Criteria 7. Was the timeframe sufficient so that one could reasonably expect to see an association between exposure and outcome if it existed? Criteria 8. For exposures that can vary in amount or level, did the study examine different levels of the exposure as related to the outcome (e.g., categories of exposure, or exposure measured as continuous variable)? Criteria 9. Were the exposure measures (independent variables) clearly defined, valid, reliable, and implemented consistently across all study participants? Criteria 10. Was the exposure(s) assessed more than once over time? Criteria 11. Were the outcome measures (dependent variables) clearly defined, valid, reliable, and implemented consistently across all study participants? Criteria 12. Were the outcome assessors blinded to the exposure status of participants? Criteria 13. Was loss to follow-up after baseline 20% or less? Criteria 14. Were key potential confounding variables measured and adjusted statistically for their impact on the relationship between exposure(s) and outcome(s)? The overall judgment is determined by Good, Fair, Poor. CD, cannot determine; NA, not applicable; NR, not reported.

Table S3. Results of the quality assessment of the study on frailty and clinical outcomes.

Author	Criteria 1	Criteria 2	Criteria 3	Criteria 4	Criteria 5	Criteria 6	Criteria 7	Criteria 8	Criteria 9	Criteria 10	Criteria 11	Criteria 12	Criteria 13	Criteria 14	Quality Rating
Patel KV et al. (2014)	Y	Y	NR	Y	N	Y	Y	NA	Y	NA	Y	NR	NR	N	Good
Krishnan M et al. (2014)	Y	N	NR	Y	N	Y	Y	NA	Y	NA	Y	NR	NR	N	Good
Kistler EA et al. (2015)	Y	Y	Y	Y	N	Y	Y	NA	N	NA	Y	NR	NR	N	Poor
Gleason LJ et al. (2017)	Y	Y	N	Y	N	Y	Y	NA	Y	NA	Y	NR	NR	N	Good
Choi JY et al. (2017)	Y	Y	NR	Y	N	Y	Y	NA	Y	NA	Y	NR	NR	N	Good
Winters AM et al. (2108)	Y	Y	NR	Y	N	Y	Y	NA	Y	NA	Y	NR	NR	N	Good
Vasu BK et al. (2018)	Y	Y	NR	Y	N	Y	Y	NA	Y	NA	Y	NR	NR	N	Poor
Chen CL et al. (2019)	Y	Y	NR	Y	N	Y	Y	NA	Y	NA	Y	NR	NR	N	Fair
Inoue T et al. (2019)	Y	Y	NR	Y	N	Y	Y	NA	Y	NA	Y	NR	NR	N	Good
Van De Ree CLP et al. (2019)	Y	Y	Y	Y	N	Y	Y	NA	Y	NA	Y	NR	N	N	Good
Jorissen RN et al. (2020)	Y	Y	NR	Y	N	Y	Y	NA	Y	NA	Y	NR	Y	N	Good
Lu W et al. (2020)	Y	Y	NR	Y	N	Y	Y	NA	N	NA	Y	NR	N	N	Poor
Pizzonia M et al. (2020)	Y	Y	Y	Y	Y	Y	Y	NA	Y	NA	Y	NR	Y	N	Good
Low S et al. (2020)	Y	Y	Y	Y	N	Y	Y	NA	Y	NA	Y	NR	Y	N	Good
Narula S et al. (2020)	Y	Y	NR	Y	N	Y	Y	NA	Y	NA	Y	NR	NR	N	Fair

Quality of the selected observational study was assessed using the National Institutes of Health (NIH) Quality Assessment tool for Observational Cohort and Cross-Sectional Studies. Criteria1. Was the research question or objective in this paper clearly stated? Criteria 2. Was the study population clearly specified and defined? Criteria 3. Was the participation rate of eligible persons at least 50%? Criteria 4. Were all the subjects selected or recruited from the same or similar populations (including the same time period)? Were inclusion and exclusion criteria for being in the study prespecified and applied uniformly to all participants? Criteria 5. Was a sample size justification, power description, or variance and effect estimates provided? Criteria 6. For the analyses in this paper, were the exposure(s) of interest measured prior to the outcome(s) being measured? Criteria 7. Was the timeframe sufficient so that one could reasonably expect to see an association between exposure and outcome if it existed? Criteria 8. For exposures that can vary in amount or level, did the study examine different levels of the exposure as related to the outcome (e.g., categories of exposure, or exposure measured as continuous variable)? Criteria 9. Were the exposure measures (independent variables) clearly defined, valid, reliable, and implemented consistently across all study participants? Criteria 10. Was the exposure(s) assessed more than once over time? Criteria 11. Were the outcome measures (dependent variables) clearly defined, valid, reliable, and implemented consistently across all study participants? Criteria 12. Were the outcome assessors blinded to the exposure status of participants? Criteria 13. Was loss to follow-up after baseline 20% or less? Criteria 14. Were key potential confounding variables measured and adjusted statistically for their impact on the relationship between exposure(s) and outcome(s)? The overall judgment is determined by Good, Fair, Poor. CD, cannot determine; NA, not applicable; NR, not reported.

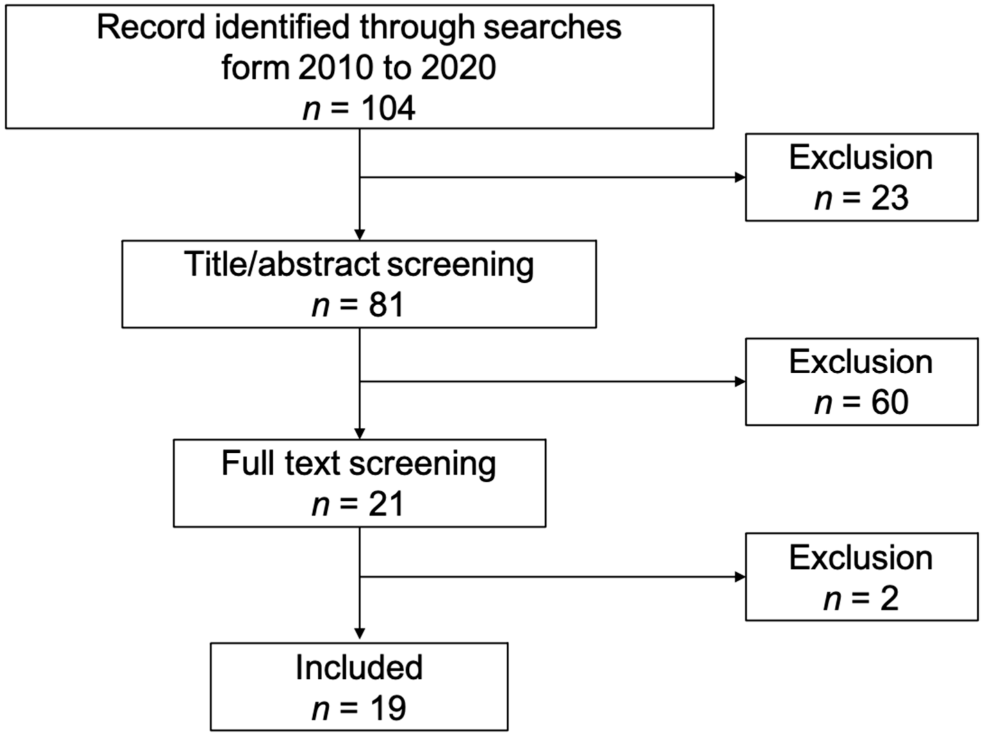

Figure S1. Flow diagrams of electronic search strategy for undernutrition with hip fractures.

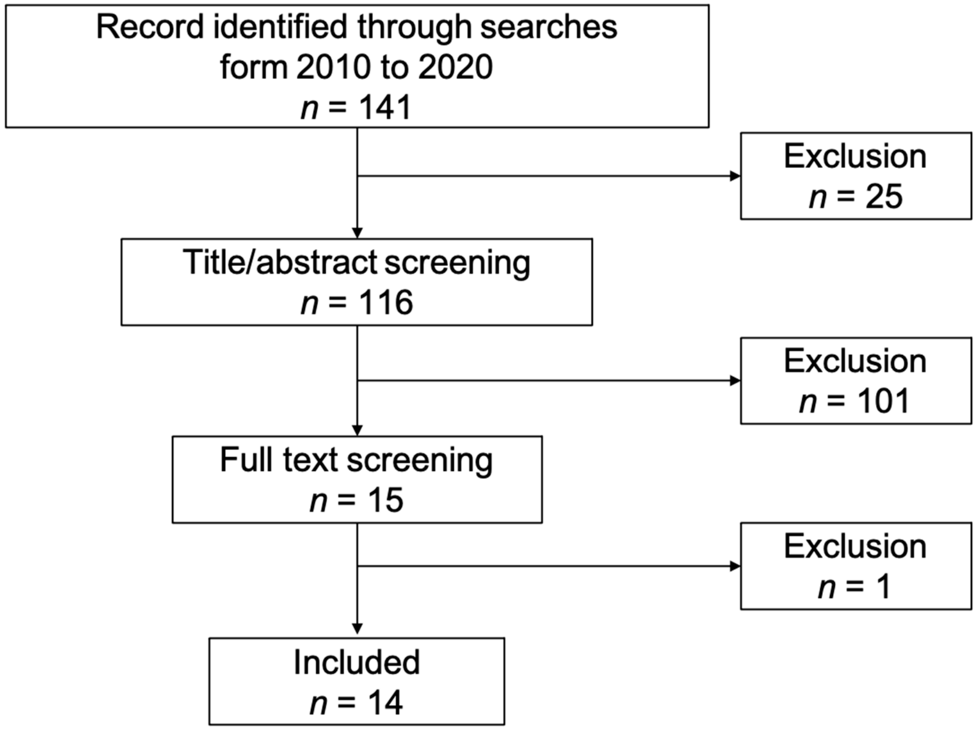

Figure S2. Flow diagrams of electronic search strategy for sarcopenia with hip fractures.

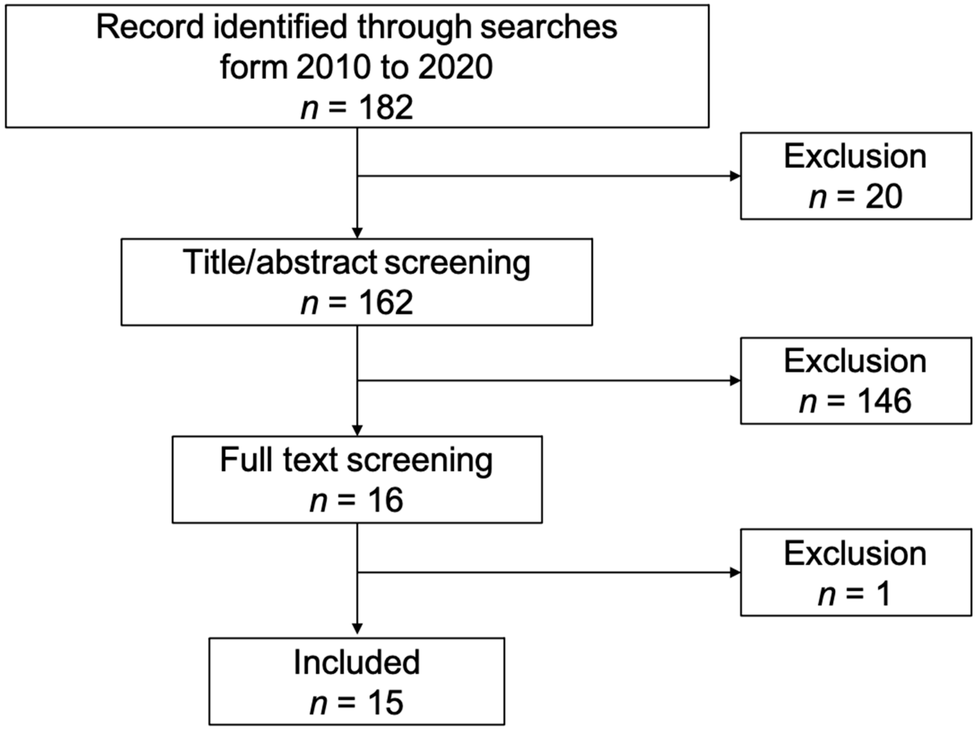

Figure S3. Flow diagrams of electronic search strategy for frailty with hip fractures.
